# Skillful prediction of hot temperature extremes over the source region of ancient Silk Road

**DOI:** 10.1038/s41598-018-25063-x

**Published:** 2018-04-27

**Authors:** Jingyong Zhang, Zhanmei Yang, Lingyun Wu

**Affiliations:** 1 Center for Monsoon System Research, Institute of Atmospheric Physics, Chinese Academy of Sciences, Beijing, 100029 China; 2 University of Chinese Academy of Sciences, Beijing, 100049 China; 3 State Key Laboratory of Numerical Modeling for Atmospheric Sciences and Geophysical Fluid Dynamics, Institute of Atmospheric Physics, Chinese Academy of Sciences, Beijing, 100029 China

## Abstract

The source region of ancient Silk Road (SRASR) in China, a region of around 150 million people, faces a rapidly increased risk of extreme heat in summer. In this study, we develop statistical models to predict summer hot temperature extremes over the SRASR based on a timescale decomposition approach. Results show that after removing the linear trends, the inter-annual components of summer hot days and heatwaves over the SRASR are significantly related with those of spring soil temperature over Central Asia and sea surface temperature over Northwest Atlantic while their inter-decadal components are closely linked to those of spring East Pacific/North Pacific pattern and Atlantic Multidecadal Oscillation for 1979–2016. The physical processes involved are also discussed. Leave-one-out cross-validation for detrended 1979–2016 time series indicates that the statistical models based on identified spring predictors can predict 47% and 57% of the total variances of summer hot days and heatwaves averaged over the SRASR, respectively. When the linear trends are put back, the prediction skills increase substantially to 64% and 70%. Hindcast experiments for 2012–2016 show high skills in predicting spatial patterns of hot temperature extremes over the SRASR. The statistical models proposed herein can be easily applied to operational seasonal forecasting.

## Introduction

Global hot temperature extremes have increased rapidly in magnitude, number and size in the past several decades^1–3^. Even during the so-called hiatus of global warming, hot temperature extremes show a continued increase with a larger tendency for the highest extreme events^4^. Extreme heat has increasingly severe impacts on human health, agriculture, natural systems, and energy consumption with high temperatures breaking many records since the beginning of the 21^st^ century^5–7^. According to the WMO report, the lives lost from extreme heat increased by 2300% in 2001–2010 compared to 1991–2000^8^. The heat-related deaths caused by each of the European heatwave of 2003 and the Russian heatwave of 2010 ran into tens of thousands^8,9^. As hot temperature extremes continue to increase, their adverse impacts on human society and the ecosystem will become more severe in the future^3,10^. However, the prediction of hot temperature extremes in advance remains very challenging, hampering our ability to prepare for and mitigate their adverse impacts.

**Figure 1:**
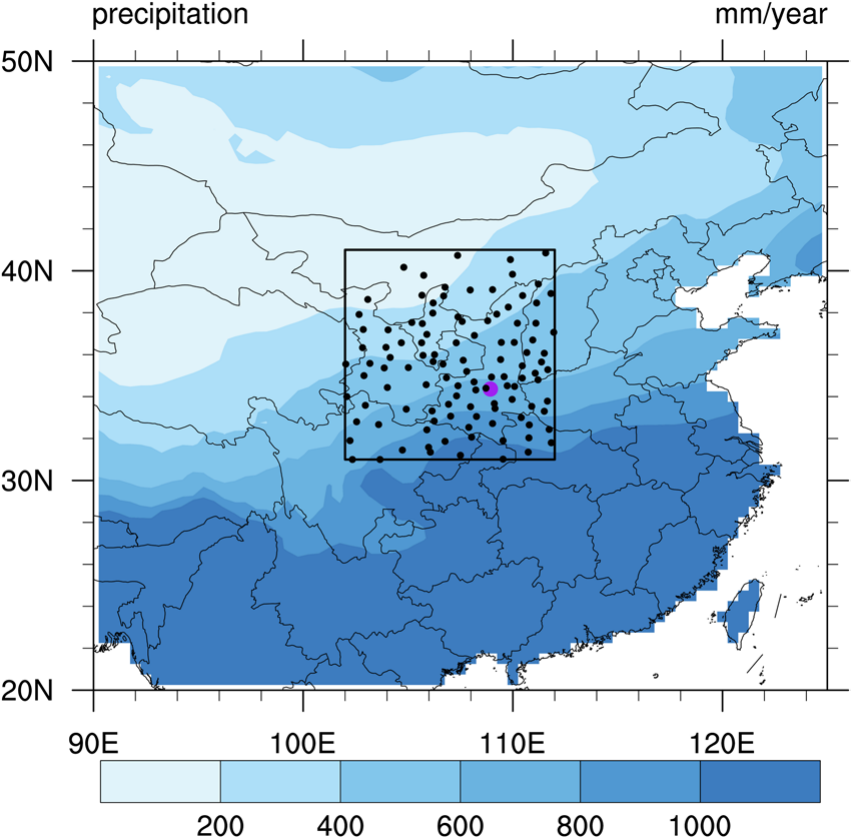
Location of stations (black dots) used in the study, analysis domain (the black box), and annual precipitation averaged over 1981–2010 from CRU TS 4.00. The purple dot denotes the location of Chang’an (Today’s Xi’an in Shaanxi province of China), the capital of Han Dynasty of China. Chang’an is the starting point of ancient Silk Road. The CRU precipitation data were obtained from University of East Anglia Climatic Research Unit (http://catalogue.ceda.ac.uk/). This map was generated with NCAR Command Language (NCL) version 6.3.0 (Boulder, Colorado: UCAR/NCAR/CISL/TDD. http://dx.doi.org/10.5065/D6WD3XH5).

The Silk Road first emerged in the Han Dynasty of China, and became a bridge connecting China with Central Asia and Europe. The starting point of the ancient Silk Road is Chang’an (today’s Xi’an in Shaanxi province of China), which is the capital of the Han Dynasty of China. The source region of the ancient Silk Road (SRASR) surrounding Xi’an is located in the transition zones between dry and wet climate (Fig. 1). This region is densely populated and ecologically fragile. The SRASR, like many other regions in the world, experienced an increasing trend in hot temperature extremes in recent decades, with substantial inter-annual and inter-decadal variations^11–14^. The occurrence of hot temperature extremes over the SRASR such as the unprecedented heatwave of 2017 can cause dramatic economic losses and severe environmental impacts^15^. Climate change projection demonstrated that future hot temperature extremes in the SRASR will become more common and more intense^16–18^. The increase of high temperature events combined with population growth and wealth accumulation poses a high heat-related disaster risk in this region^15,19^.

Observational and modeling studies have demonstrated that soil moisture and temperature conditions can largely influence daily maximum temperature, and thus is of critical importance to the occurrence of hot temperature extremes^20–25^. In addition, the slowly evolving sea surface temperature (SST) and large-scale circulation patterns such as El Niño-Southern Oscillation (ENSO) and the Atlantic Multidecadal Oscillation (AMO) have long been known to play important roles in influencing East Asian summer temperature, and associated temperature extremes^26–35^.

The anomalies of soil moisture and temperature, SST, and large-scale circulation patterns can persist from one month to several years, and may thus provide key potential predictors for hot temperature extremes over the SRASR and other regions of the globe. The objective of this study is to investigate the role of soil moisture and temperature conditions, SST, and large-scale circulation patterns in spring (March–April–May) for the prediction of summer (June–July–August) hot temperature extremes over the SRASR [102°E–112°E, 31°N–41°N] enclosed by the black box in Fig. 1 for the period of 1979–2016.

A hot day is defined as the one with daily maximum surface air temperature meeting or exceeding 35 °C, and a heatwave event is defined as a spell of at least two consecutive hot days. We firstly remove the linear trends of summer hot days and heatwaves averaged over the SRASR for 1979–2016, and decompose detrended time series into the inter-annual and inter-decadal components (see Data and Methods for details). We further identify spring predictors for the inter-annual and inter-decadal components of summer hot temperature extremes over the SRASR, and discuss the possible physical mechanisms. Based on these identified predictors, the statistical models are developed for the inter-annual and inter-decadal components, and are taken together to predict detrended summer hot temperature extremes over the SRASR. Then, the linear trends are added to both observed and predicted detrended 1979–2016 time series of summer hot temperature extremes over the SRASR. Leave-one-out cross-validation is applied to test the capability of the statistical models to predict 1979–2016 time series of summer hot temperature extremes over the SRASR. Finally, we develop the statistical models using these identified predictors for each station of the SRASR, and perform hindcast experiments to predict spatial patterns of summer hot temperature extremes over the SRASR for 2012–2016.

## Results

### Relationship of summer hot temperature extremes over the SRASR to spring soil moisture and temperature conditions

**Figure 2:**
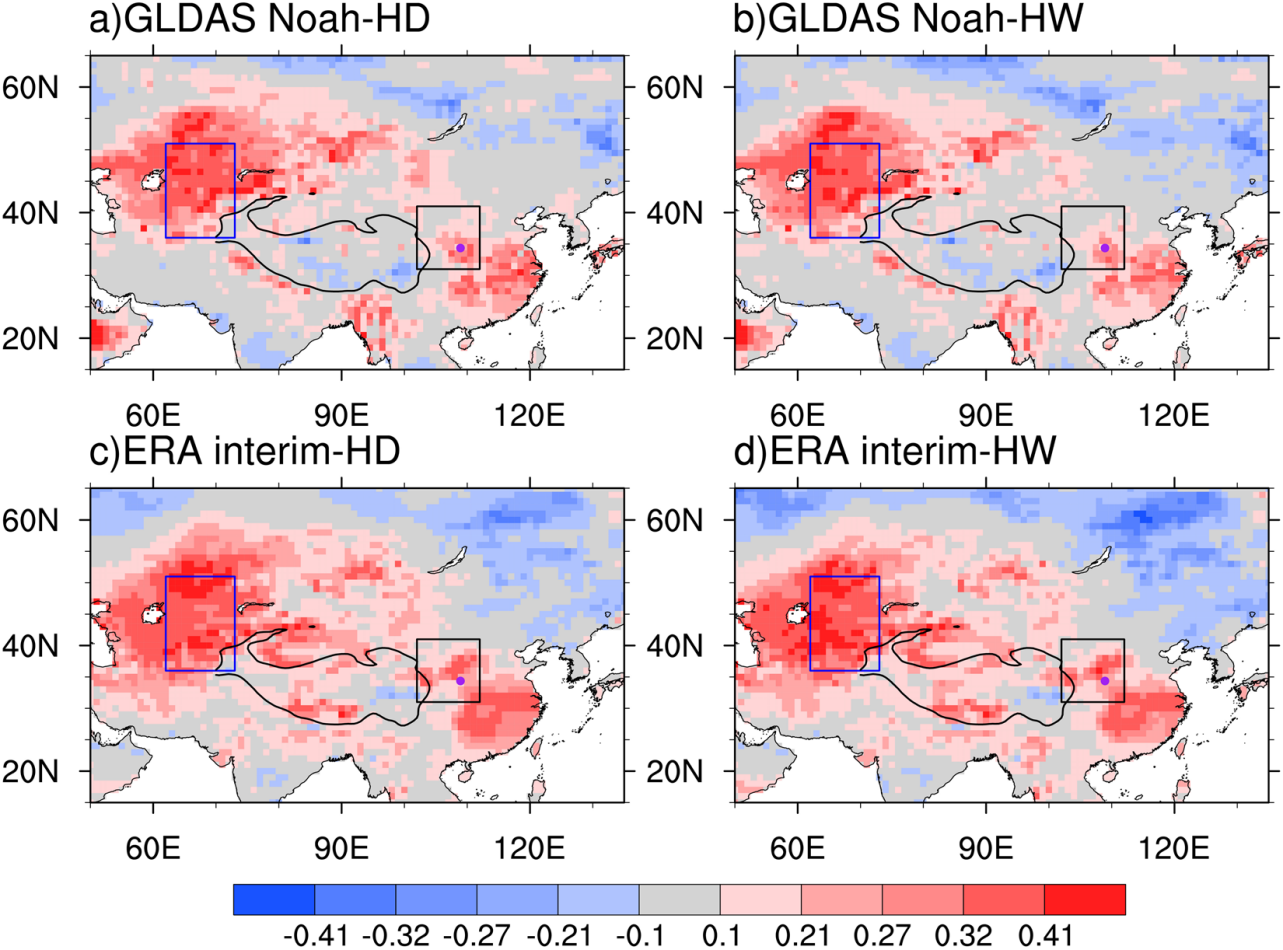
Correlation pattern of summer hot days (left panel) and heatwaves (right panel) averaged over the SRASR (source region of ancient Silk Road) enclosed by the black box with preceding spring soil temperature from (**a**,**b**) GLDAS-Noah and (**c,d**) ERA-interim for the period of 1979–2016. All data are detrended before correlation coefficients are calculated. The blue box represents the key region of soil temperature [62°E–73°E, 36°N–51°N], which is located in Central Asia, and the black solid line denotes topographic contour line of 3000 m. Correlations of ±0.27, ±0.32 and ±0.41 indicate the 90%, 95% and 99% significance levels. This map was generated with NCAR Command Language (NCL) version 6.3.0 (Boulder, Colorado: UCAR/NCAR/CISL/TDD. http://dx.doi.org/10.5065/D6WD3XH5).

**Figure 3:**
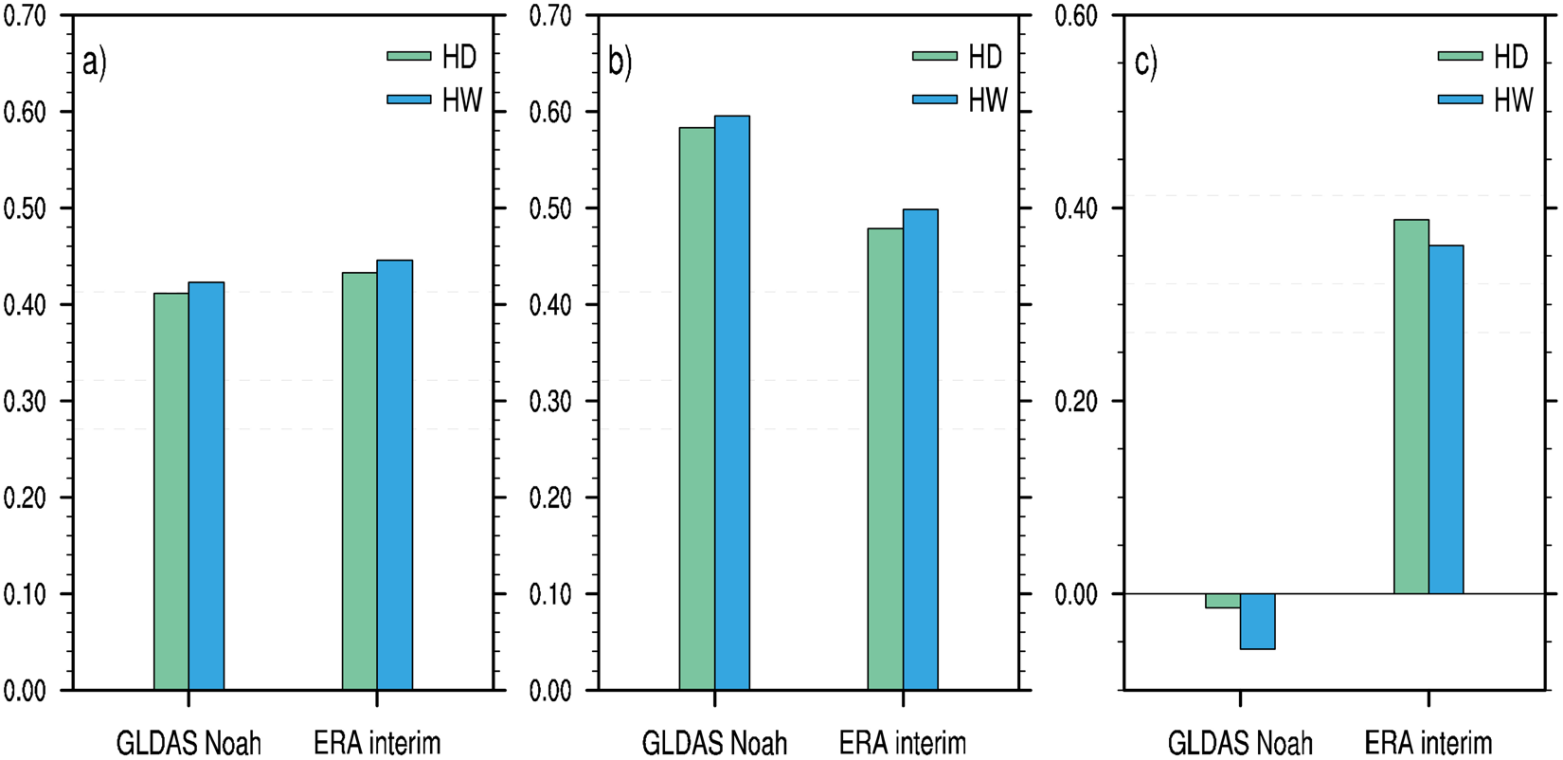
Correlation coefficients of spring soil temperature averaged over Central Asia in GLDAS-Noah and ERA-interim with summer hot days (HD) and heatwaves (HW) averaged over the SRASR (source region of ancient Silk Road) for the period of 1979-2016: (**a**) detrended time series, (**b**) inter-annual components of detrended time series; (**c**) inter-decadal components of detrended time series. Correlations of ±0.27, ±0.32 and ±0.41 indicate the 90%, 95% and 99% significance levels (the dash lines).

After removing the linear trends, we examine the correlations of summer hot temperature extremes averaged over the SRASR with preceding spring soil moisture and temperature conditions for the period of 1979–2016. There are no consistent areas in which significant correlations of summer hot temperature extremes averaged over the SRASR with spring soil moisture exist for GLDAS-Noah and ERA-interim datasets (Fig. S1). In comparison, consistent and strong correlations with spring soil temperature appear over Central Asia in both GLDAS-Noah and ERA-interim datasets (Fig. 2). We further calculate correlation coefficients between spring soil temperature averaged over Central Asia enclosed by the blue box in Fig. 2 [62°E–73°E, 36°N–51°N] and summer hot temperature extremes averaged over the SRASR (Fig. 3a). The correlations of summer hot days and heatwaves over the SRASR to spring soil temperature over Central Asia in GLDAS-Noah and ERA-interim range from 0.41 to 0.45, which are all significant at the 99% confidence level. The agreements in GLDAS-Noah and ERA-interim indicate the robustness of the close relationship between spring soil temperature over Central Asia and summer hot temperature extremes over the SRASR. The inter-annual components of spring soil temperature over Central Asia in both GLDAS-Noah and ERA-interim datasets are highly correlated with those of summer hot temperature extremes over the SRASR while there are no consistent significant correlations for the inter-decadal components (Fig. 3b,c). We therefore identify the inter-annual components of spring soil temperature over Central Asia as a predictor for those of summer hot temperature extremes over the SRASR. As the correlations are higher in GLDAS-Noah than ERA-interim, GLDAS-Noah soil temperature is used in statistical prediction models.

We further explore the possible physical processes underlying the close link of the inter-annual component of spring soil temperature condition over Central Asia to summer hot temperature extremes over the SRASR. According to warmer years of the inter-annual component of spring soil temperature over Central Asia, there are positive 500 hPa geopotential height anomalies over northern china, which may result in more downward solar radiation and increased subsidence warming (Fig. S2a). In addition, the warm advection from the south to the SRASR tends to be enhanced (Fig. S2b). These changes in regional atmospheric circulation features tend to warm surface air temperatures, and lead to more hot temperature extremes over the SRASR. There is strong evidence that the western North Pacific subtropical high (WNPSH) can largely influence hot temperature extremes over China^36–38^. The north-westward extension of the WNPSH associated with warmer spring soil temperature over Central Asia on its inter-annual component may contribute to the occurrence of hot temperature extremes over the SRASR.

### Relationship of summer hot temperature extremes over the SRASR to spring SST

**Figure 4:**
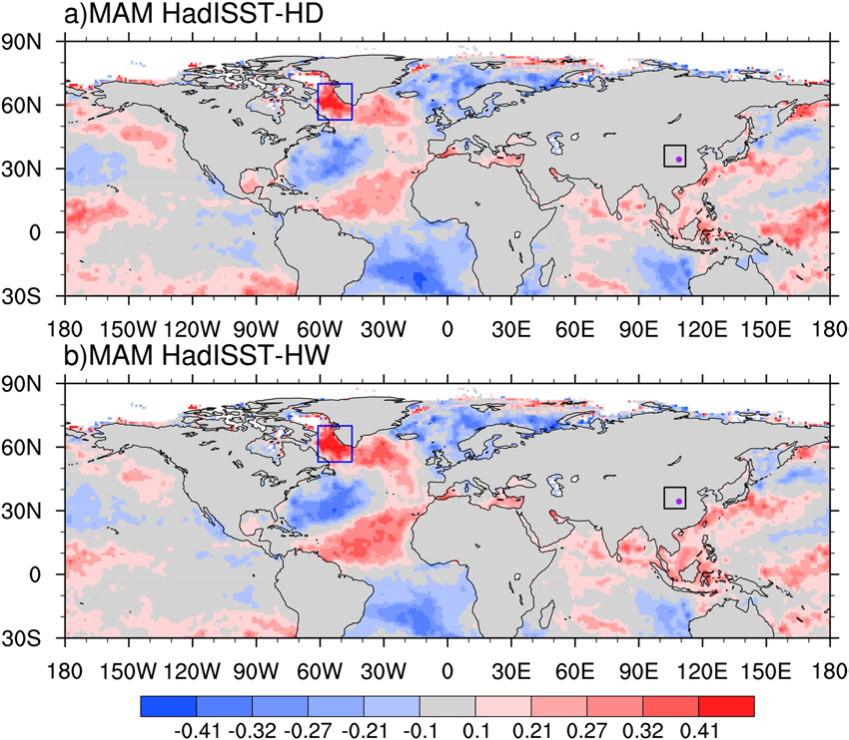
Correlation pattern of summer (**a**) hot days and (**b**) heatwaves averaged over the SRASR (source region of ancient Silk Road) enclosed by the black box with preceding spring sea surface temperature (SST) for the period of 1979-2016. All data are detrended before correlation coefficients are calculated. The blue box represents the key region of SST [45°W–61°W, 53°N–70°N], which is located in Northwest Atlantic. Correlations of ±0.27, ±0.32 and ±0.41 indicate the 90%, 95% and 99% significance levels. This map was generated with NCAR Command Language (NCL) version 6.3.0 (Boulder, Colorado: UCAR/NCAR/CISL/TDD. http://dx.doi.org/10.5065/D6WD3XH5).

**Figure 5:**
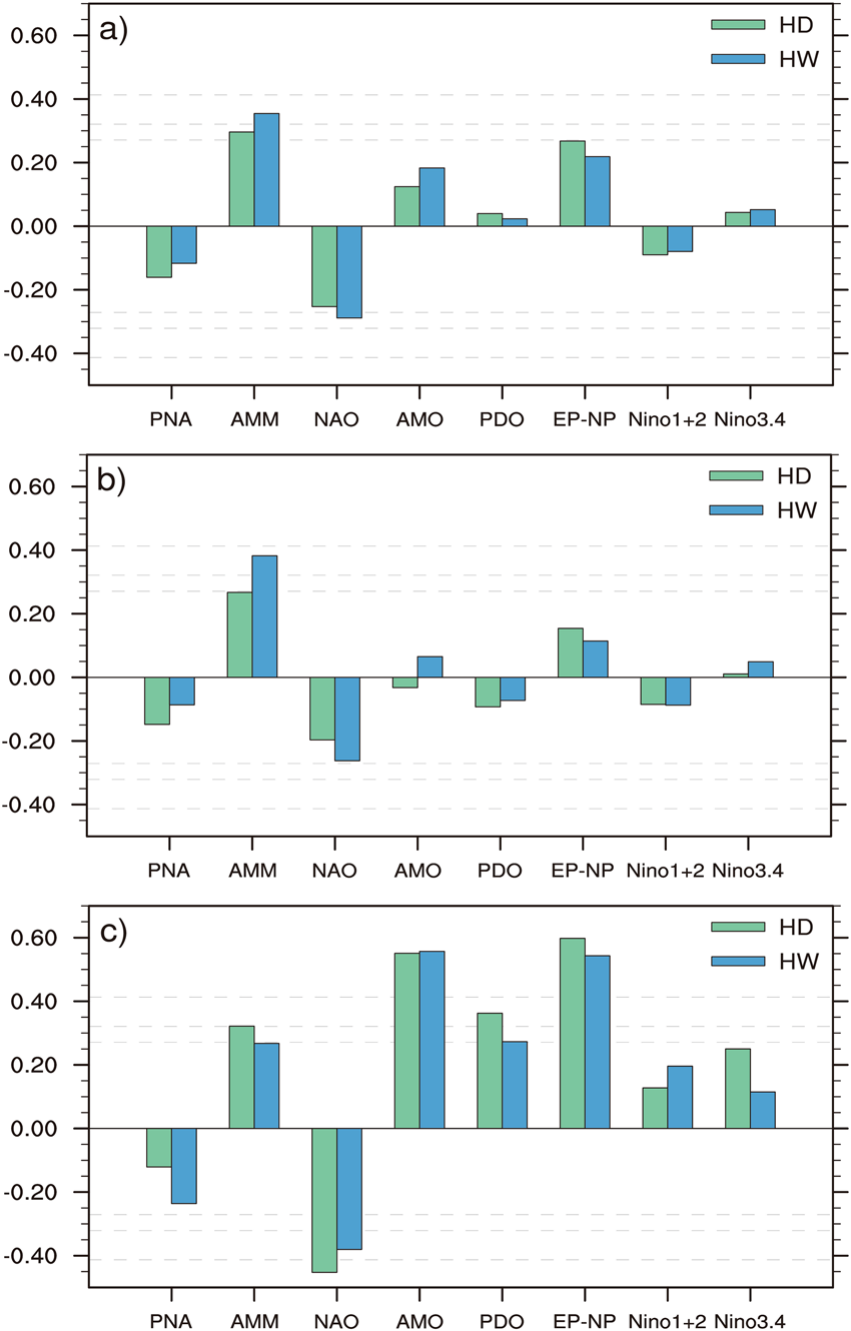
Correlation coefficients of spring large-scale climate indices with summer hot days (HD) and heatwaves (HW) averaged over the SRASR (source region of ancient Silk Road) for the period of 1979–2016: (**a**) detrended time series, (**b**) inter-annual components of detrended time series, (**c**) inter-decadal components of detrended time series. The climate indices used in this study include PNA, AMM, NAO, AMO, PDO, EP-NP, Nino1 + 2, and Nino3.4. Correlations of ±0.27, ±0.32 and ±0.41 indicate the 90%, 95% and 99% significance levels (the dash lines).

Figure 4 presents the correlations of summer hot days and heatwaves averaged over the SRASR with preceding spring SST for the period of 1979–2016. The strongest correlations appear over Northwest Atlantic. Warmer spring SST over this region is associated with the increased summer hot days and heatwaves over the SRASR. We average spring SST over Northwest Atlantic enclosed by the blue box in Fig. 4 [45°W–61°W, 53°N–70°N], and calculate its correlations with summer hot temperature extremes averaged over the SRASR. The correlation coefficients with summer hot days and heatwaves are 0.45 and 0.48, both significant at the 99% confidence level (Fig. S3). We therefore identify Northwest Atlantic as the key region of spring SST, which can be used as a potential source of seasonal prediction of summer hot temperature extremes over the SRASR. The correlations of spring SST averaged over the key region with those over North Atlantic show a clear tripole structure with high and positive values appearing over the northern part (North of 50°N) (Fig. S4). This indicates that the close relationship of spring SST over Northwest Atlantic with summer hot temperature extremes over the SRASR may largely reflect the effects of North Atlantic SST tripole^39^. The inter-annual and inter-decadal components of spring SST over Northwest Atlantic both correlate significantly with those of summer hot temperature extremes over the SRASR (Fig. S3). We choose the inter-annual component of spring SST over Northwest Atlantic as a predictor for those of summer hot temperature extremes over the SRASR. The inter-decadal component of spring SST is not used as the predictor for those of summer hot temperature extremes over the SRASR since there are stronger correlations with those of the spring the East Pacific/North Pacific Oscillation (EP-NP) pattern and the spring AMO (Fig. 5).

Composite difference of summer 500-hPa geopotential heights between the ten warmest and ten coolest years of spring SST’s inter-annual component shows that warmer spring SST anomalies over Northwest Atlantic for the inter-annual component are associated with positive anomalies in summer 500-hPa geopotential height over Mongolia and northern China including the SRASR [Fig. S5a]. The positive summer 500-hPa geopotential height anomalies may result in more downward solar radiation accompanied by more clear skies as well as stronger subsidence warming and reduced precipitation, and thus favor the occurrence of hot temperature extremes over the SRASR. Composite analysis of summer 850-hPa wind vector shows that warmer spring SST over Northwest Atlantic on its inter-annual component tends to increase the southerlies over western part of the SRASR, and thus enhance the warm advection from the south to these areas (Fig. S5b).

### Relationship of summer hot temperature extremes over the SRASR to spring large-scale climate patterns

Figure 5 presents the correlations between summer hot temperature extremes averaged over the SRASR and preceding spring large-scale climate patterns including the Pacific North American Index (PNA), the Atlantic Meridional Mode (AMM), the North Atlantic Oscillation (NAO), the AMO, the Pacific Decadal Oscillation (PDO), the EP-NP, Nino1 + 2, and Nino3.4 based on detrended 1979–2016 time series, and their inter-annual and inter-decadal components. Summer hot days and heatwaves averaged over the SRASR generally have insignificant correlations with spring large-scale climate indices regarding detrended time series and their inter-annual components at the 95% confidence level (Fig. 5a,b). The inter-decadal components of summer hot days and heatwaves over the SRASR have high correlations with those of two large-scale phenomena: the spring EP-NP pattern and the spring AMO (Fig. 5c). The correlation coefficients with the spring EP-NP pattern and the spring AMO for the inter-decadal components range from 0.54 to 0.60. In comparison to the spring EP-NP pattern, the spring AMO has slightly lower and higher correlations with summer hot days and heat waves over the SRASR, respectively.

We subsequently examine regional atmospheric circulation features associated with the inter-decadal components of the spring EP-NP pattern and the spring AMO. Composite analysis shows that the positive phase of the spring EP-NP pattern on its inter-decadal component is associated with increased summer 500-hPa geopotential heights over East Asia, which tend to warm surface temperatures and lead to more hot temperature extremes over the SRASR through the enhancement of downward solar radiation and subsidence warming (Fig. S6a). The positive phase of the spring AMO on its inter-decadal component tends to result in the increased summer 500 hPa geopotential heights over Mongolia and northern China, and favors more hot temperature extremes over the SRASR (Fig. S7a). In addition, the increased easterlies associated with the positive phase of the spring AMO on its inter-decadal component can advect more warm air to the SRASR, and thus provide the condition for extreme high temperatures to occur over this region (Fig. S7b).

### Prediction of summer hot temperature extremes over the SRASR

As described above, the inter-annual components of detrended time series of summer hot days and heatwaves averaged over the SRASR have close relations with those of spring soil temperature over Central Asia ($MAM\_Soil{T}_{ia}$) and spring SST over Northwest Atlantic ($MAM\_SS{T}_{ia}$), while their inter-decadal components are highly correlated with those of the spring EP-NP pattern ($MAM\_EPN{P}_{id}$) and the spring AMO ($MAM\_AM{O}_{id}$) for the period of 1979–2016. The correlations between $MAM\_Soil{T}_{ia}$ and $MAM\_SS{T}_{ia}$ (r = 0.03) and between $MAM\_EPN{P}_{id}$ and $MAM\_AM{O}_{id}$ (r = −0.26) are both small and insignificant.

We further establish the statistical models for the two different time components of summer hot temperature extremes over the SRASR by linearly regressing the inter-annual components of detrended 1979–2016 time series onto $MAM\_Soil{T}_{ia}$ and $MAM\_SS{T}_{ia}$, and the inter-decadal components onto $MAM\_EPN{P}_{id}$ and $MAM\_AM{O}_{id}$:

1 $${H}_{ia}={f}_{a}({\rm{MAM}}\_{{\rm{SoilT}}}_{{\rm{ia}}},{\rm{MAM}}\_{{\rm{SST}}}_{{\rm{ia}}})$$

2 $${H}_{id}={f}_{d}(MAM\_EPN{P}_{id},MAM\_AM{O}_{id})$$

${H}_{ia}$ and ${H}_{id}$ represent the inter-annual and inter-decadal components of summer hot temperature extremes. Take them together, the detrended time series of summer hot temperature extremes over the SRASR can be obtained.

3 $${{\rm{HD}}}_{{\rm{d}}}=H{D}_{ia}+H{D}_{id}$$

4 $${{\rm{HW}}}_{{\rm{d}}}=H{W}_{ia}+H{W}_{id}$$

HD and HW represent hot days and heatwaves, respectively. The linear trends of HD and HW can be obtained as follows.

5 $${{\rm{HD}}}_{{\rm{t}}}=3.240+0.137\times (iyear-1979)$$

6 $${{\rm{HW}}}_{{\rm{t}}}=0.752+0.0284\times (iyear-1979)$$

iyear represents the predicted year. We further put the linear trends back to obtain predicted time series of hot days and heat waves.

7 $${\rm{HD}}=H{D}_{t}+H{D}_{d}$$

8 $${\rm{HW}}=H{W}_{t}+H{W}_{d}$$

We leave the same one year out from the 1979–2016 time series for both inter-annual and inter-decadal components each time, and use the data from the remaining years as the training set. We repeat this process until the predictions for all years are performed. Doing so allows us to use leave-one-out cross-validation to inter-annual, inter-decadal and whole time series of summer hot temperature extremes over the SRASR.

For inter-annual components of detrended 1979–2016 time series, the correlation coefficients between observed and predicted values for hot days and heatwaves over the SRASR are 0.65 and 0.74 when $MAM\_Soil{T}_{ia}$ and $MAM\_SS{T}_{ia}$ are both used (Fig. S8a,b). The correlation coefficients between the observations and predictions for the inter-annual components of summer hot days and heatwaves are 0.52 and 0.54 with using $MAM\_Soil{T}_{ia}$ alone, and 0.37 and 0.48 with using $MAM\_SS{T}_{ia}$ alone. For the inter-decadal components with using both $MAM\_EPN{P}_{id}$ and $MAM\_AM{O}_{id}$, the correlation coefficients between the observations and predictions for hot days and heatwaves are 0.93 and 0.88 (Fig. S8c,d). Using $MAM\_EPN{P}_{id}$ alone yields correlation coefficients of 0.55 and 0.49 for hot days and heatwaves, while they are both 0.49 with using $MAM\_AM{O}_{id}$ alone.

**Figure 6:**
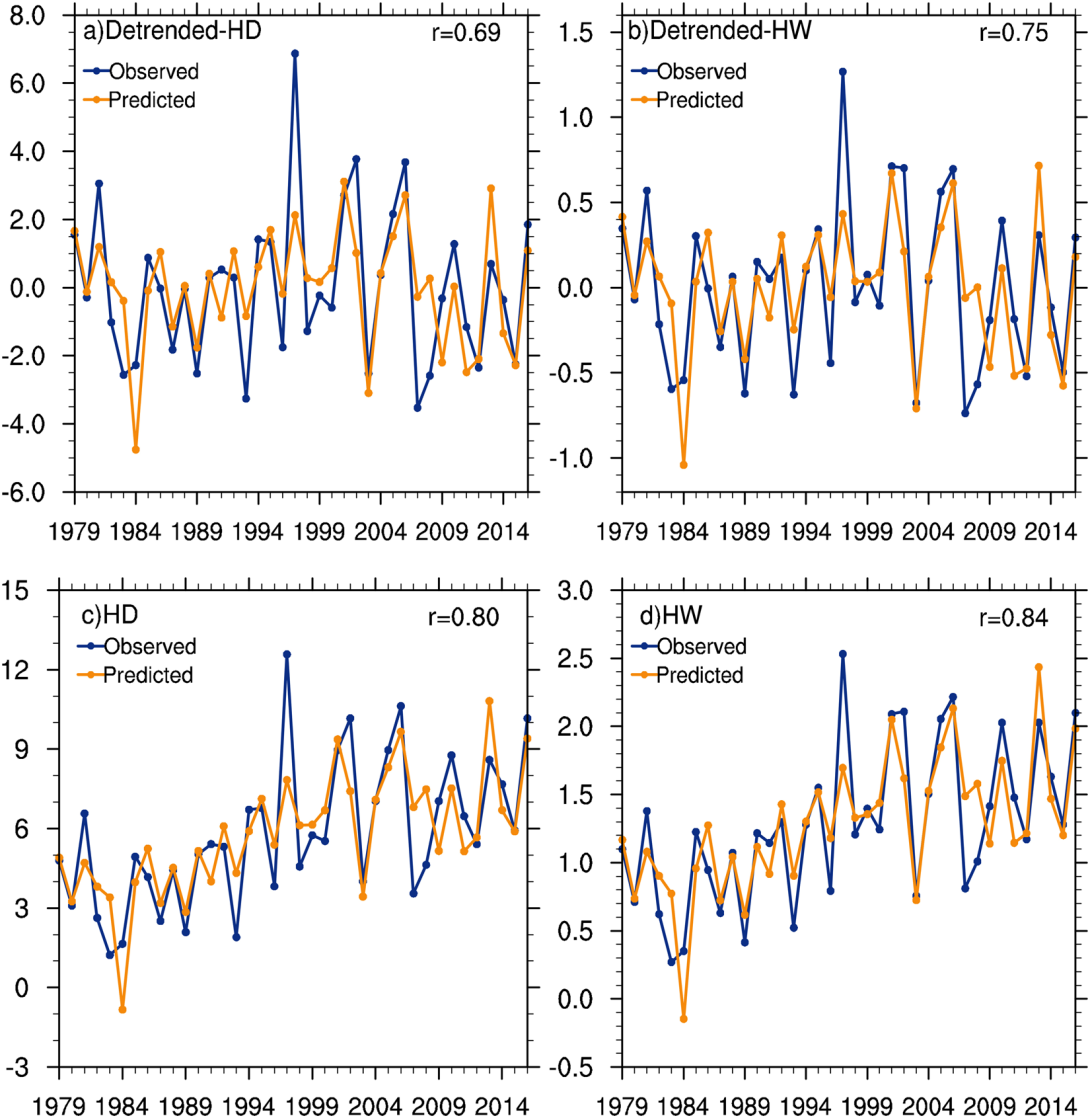
Time series of observed and predicted summer hot temperature extremes averaged over the SRASR (source region of ancient Silk Road) without (upper panel) and with (lower panel) linear trends included for the period of 1979–2016: (**a,c**) hot days, (**b,d**) heatwaves. The correlation coefficients between the observations and predictions are also shown.

For the whole detrended 1979–2016 time series as the sum of inter-annual and inter-decadal components, the statistical models yield correlations of 0.69 and 0.75 between observed and predicted values for hot days and heatwaves (Fig. 6a,b). These results mean that 47% and 57% of the total variances of summer hot days and heatwaves averaged over the SRASR can be predicted by the statistical models. When the linear trends are put back, the predicted total variances of the summer hot days and heatwaves substantially increase to 64% and 70% (Fig. 6c,d).

**Figure 7:**
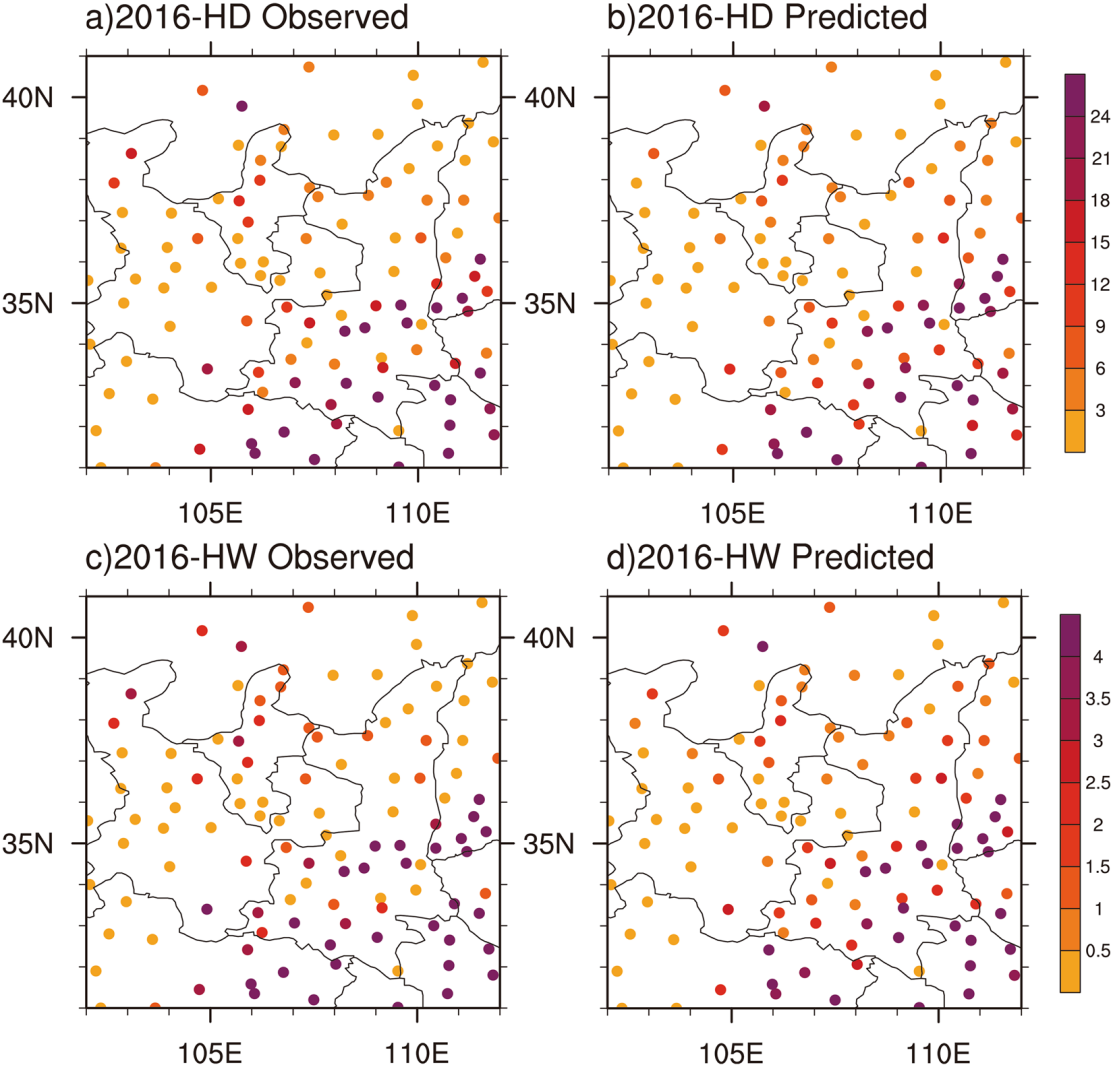
Spatial patterns of observed (left panel) and predicted (right panel) summer hot temperature extremes over the SRASR (source region of ancient Silk Road) in 2016: (**a**,**b**) hot days; (**c**,**d**) heat waves.

**Figure 8:**
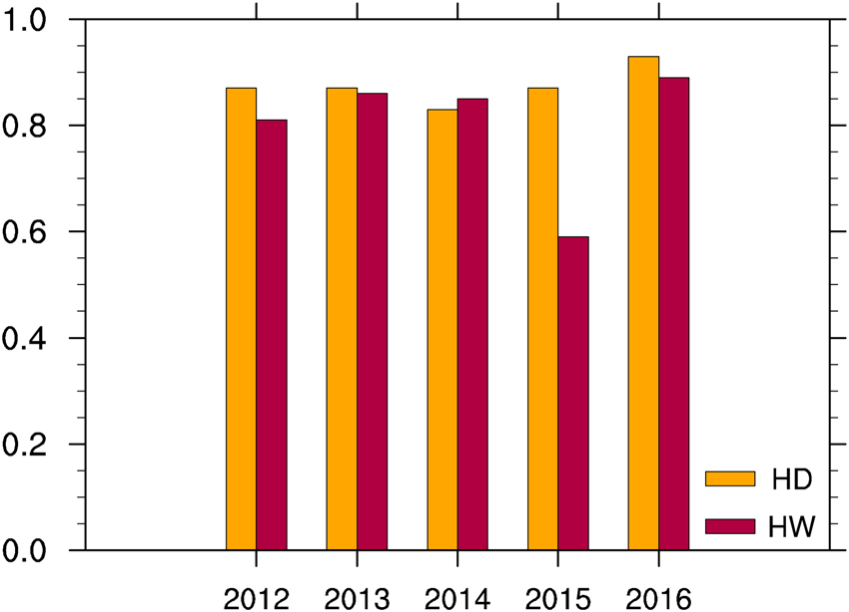
Correlation coefficients of spatial patterns of observed and predicted summer hot days (HD) and heatwaves (HW) at 111 stations of the SRASR (source region of ancient Silk Road) for 2012–2016.

Finally, we use those previously identified spring predictors to develop statistical models to predict spatial patterns of summer hot days and heatwaves over the SRASR for 2012–2016. Based on the timescale decompostion approach, the statistical models are developed with detrended time series of summer hot temperature extremes at each station and these previously identified predictors for 1979–2011, 1979–2012, 1979–2013, 1979–2014, and 1979–2015 to predict summer hot temperature extremes of 2012, 2013, 2014, 2015 and 2016, respectively. The linear trends are further added to the detrended values, and the correlation coefficients of spatial patterns are calculated to test the prediction skills. As an example, Fig. 7 presents the observed and predicted summer hot days and heatwaves for all 111 stations over the SRASR in 2016. The clear southeast-to-northwest gradients of summer hot temperature extremes over the SRASR in 2016 are well predicted by the statistical models. For 2012–2016, spatial correlation coefficients are all higher than 0.80 except for heatwaves of 2015 (Fig. 8).

These results together indicate that the statistical models proposed in this study have high skills in predicting summer hot days and heatwaves over the SRASR in terms of both temporal changes and spatial patterns. We also define a heatwave event as a spell of three or more consecutive hot days, and repeat our analyses and predictions. It is found that the results are quite consistent with those with a heatwave event defined as at least two consecutive hot days.

## Conclusions and Discussion

Hot temperature extremes severely influence human society and the ecosystem. However, seasonal prediction of hot temperature extremes remains very challenging. The slowly varying variables of the climate system such as soil moisture and temperature conditions, SST, and large-scale circulation patterns may contain key predictive information for hot temperature extremes. The SRASR in China, a region of around 150 million people, faces a rapidly increased heat-related disaster risk in the future. In this study, we find that the inter-annual components of detrended 1979–2016 time series of summer hot days and heatwaves over the SRASR are closely related with those of spring soil temperature over Central Asia and spring SST over Northwest Atlantic while their inter-decadal components have high correlations with those of the spring EP-NP pattern and the spring AMO.

The possible physical mechanisms are further discussed. Changes in soil temperature over Central Asia, SST over Northwest Atlantic, the EP-NP pattern and the AMO in spring have remote effects on regional circulation features, and thus may subsequently influence summer hot temperature extremes over the SRASR. Warmer spring soil temperature over Central Asia and spring SST over Northwest Atlantic on their inter-annual components, and the positive phases of the spring EP-NP pattern and the spring AMO on their inter-decadal components tend to strengthen regional geopotential heights and/or enhance advection of warm air, and thus favor summer hot temperature extremes over the SRASR, and vice versa. It is proposed that SST over Northwest Atlantic, the EP-NP pattern and the AMO in spring can modulate regional atmospheric circulation conditions over the SRASR and surrounding areas through circumglobal teleconnection pattern or Rossby wave activities, as demonstrated in previous studies^34,40,41^.

We develop the statistical models to predict summer hot temperature extremes over the SRASR for the period of 1979–2016 based on these identified spring predictors. Leave-one-out cross-validation indicates that the statistical models predict 47% and 57% of the total variances of detrended 1979–2016 time series of summer hot days and heatwaves averaged over the SRASR. When the linear trends are included, the statistical models predict substantially higher variances of 64% and 70%. In addition, hindcast experiments for 2012–2016 show that spatial patterns of summer hot temperature extremes over the SRASR can be highly predicted. The prediction can be made in June with spring averaged soil temperature condition, SST and large-scale circulation patterns, or earlier if the predictors averaged in earlier months are used. As an example, Fig. S9 shows that the statistical models still yield high prediction skills, when using the same predictors but for March-April averages.

Some uncertainties and limitations of this study remain, and warrant further investigation. Due to different daytime and nighttime surface energy budgets, daytime and nighttime surface air temperatures over the SRASR may be asymmetrically affected by land surface conditions, SST and large-scale circulation patterns, which can result in asymmetric predictability of hot temperature extremes associated with daily maximum and minimum surface air temperatures. In this study, we focus on the prediction of summer hot temperature extremes based on daily maximum surface air temperature over the SRASR. In the future, the predictions of summer hot temperature extremes associated with daily minimum surface air temperature and combined daily maximum and minimum surface air temperature conditions over this region should be performed. The proposed physical mechanisms underlying these links of summer hot temperature extremes over the SRASR to spring predictors need to be better understood by using model experiments. We use linear statistical models to predict summer hot temperature extremes, yet many nonlinear processes are involved in seasonal climate anomalies^42^. In the future, dynamical models should be further used for seasonal prediction of summer hot temperature extremes over the SRASR. Then, the predictions by statistical and dynamical models can be combined, and may therefore better enhance the prediction skills of summer hot temperature extremes over this region.

In addition to soil moisture and temperature conditions, SST, and large-scale circulation patterns, other long memory drivers such as snow cover, vegetation greenness, and sea ice can provide potentially useful predictive information^43–45^. The prediction skills of summer hot temperature extremes over the SRASR could be improved if these additional factors are appropriately taken into account. In addition, solar activity, large volcanic eruptions, and human-induced changes in concentrations of greenhouse gases and aerosols, and land use conditions also play roles in influencing summer temperature variations^46–50^. The linear trends of summer hot temperature extremes over the SRASR may largely reflect the effects of human-induced global warming. Meanwhile, it should be noted that there are large difficulties to appropriately consider anthropological impacts in statistical prediction models. Nevertheless, our findings highlight the utility and usability of antecedent land surface condition, SST, and large-scale climate indices for seasonal prediction of summer hot temperature extremes with the SRASR as an example. The statistical models proposed in this study are easily implemented into operational prediction, and the skill obtained using purely statistical technique may provide a baseline of level for future dynamical forecasting.

## Data and Methods

### Data

For daily maximum surface air temperature, we use the data at 824 observational stations in China which were obtained from the China Meteorological Data Service Center of the China Meteorological Administration (http://data.cma.cn/). This dataset was quality-controlled, processed, and released by the National Meteorological Information Center of the China Meteorological Administration. We remove the stations which have any missing data during summers of 1979–2016, and use 111 stations in the SRASR (Fig. 1).

Due to the scarcity of the observational data, we use subsurface soil moisture and temperature data from Noah of Global Land Data Assimilation System (GLDAS) version 1.0 with soil layer of 40–100 cm for the period of 1979–2016^51^. To test the robustness of our results, we also use subsurface soil moisture and temperature data from the ERA-interim provided by the European Centre for Medium-Range Weather Forecasts (ECMWF) with soil layer of 28–100 cm^52^. The soil moisture and temperature data in both GLDAS-Noah and ERA-interim validate well against the available limited observations^53,54^.

For SST, we use the Hadley Centre Sea Ice and Sea Surface Temperature dataset (HadISST) Version 1.1 for the period of 1979–2016^55^. Large-scale climate indices used in this study include the Pacific North American Index (PNA), the Arctic Oscillation (AO), the North Atlantic Oscillation (NAO), the Atlantic Multidecadal Oscillation (AMO), the Pacific Decadal Oscillation (PDO), Nino1 + 2, Nino3.4, the Atlantic Meridional Mode (AMM), and the East Pacific/North Pacific Oscillation (EP-NP). All climate indices were obtained from NOAA ESRL Physical Sciences Division (https://www.esrl.noaa.gov/psd/). The 500 hPa geopotential height and 850 hPa wind vector and temperature data from the ERA-interim reanalysis^52^ are used to investigate the possible physical mechanisms underlying the relationships of hot temperature extremes over the SRASR to spring predictors.

### Methods

Correlation and composite analyses are applied to identify potentially useful preceding spring factors for the prediction of summer hot temperature extremes over the SRASR, and explore the possible physical processes explaining these links. Before correlation and composite analyses are conducted, the linear trends of all data are removed. Since there are substantial inter-annual and inter-decadal variations in hot temperature extremes over the SRASR, Fourier decomposition filtering approach is used to decompose the detrended time series of hot days or heat waves averaged over the SRASR into the inter-annual and inter-decadal components. The preceding spring predictors are identified for the inter-annual and inter-decadal components of summer hot temperature extremes over the SRASR. Based on the identified spring predictors, we further develop different statistical models to predict two different time components of hot days and heat waves over the SRASR. Take the two time components together, the detrended time series can be obtained. Then, the linear trends of summer hot days and heatwaves are put back to both observed and predicted detrended time series. Leave-one-out cross-validation is adopted to test the capability of the statistical models to predict summer hot temperature extremes over the SRASR. The predicted variances of summer hot temperature extremes explained by the statistical models can be obtained by calculating the square of the correlation coefficients of observed time series with the corresponding cross-validation estimates. Finally, we develop statistical models for the inter-annual and inter-decadal components of detrended time series of summer hot temperature extremes at each station of the SRASR using previously identified spring predictors based on the periods of 1979–2011, 1979–2012, 1979–2013, 1979–2014 and 1979–2015 to predict summer hot temperature extremes at each station in 2012, 2013, 2014, 2015 and 2016, respectively. The linear trends are put back, and spatial correlation coefficients are calculated to test the skills of statistical models in predicting spatial patterns of hot temperature extremes over the SRASR.

## Electronic supplementary material

Supporting Figures
