# Supplementary material for: Skillful prediction of hot temperature extremes over the source region of ancient Silk Road
Source: Sci Rep. 2018 Apr 27;8:6677. doi: 10.1038/s41598-018-25063-x (PMC5923271; doi:10.1038/s41598-018-25063-x)
Supplement: Supplementary file 1 — Supporting Figures [file 41598_2018_25063_MOESM1_ESM.pdf]

Skillful prediction of hot temperature extremes over the source region of ancient Silk Road

Jingyong Zhang[1,2], Zhanmei Yang[1,2], and Lingyun Wu[3]

[1]Center for Monsoon System Research, Institute of Atmospheric Physics, Chinese Academy of Sciences, Beijing 100029, China

[2]University of Chinese Academy of Sciences, Beijing 100049, China

[3]State Key Laboratory of Numerical Modeling for Atmospheric Sciences and Geophysical Fluid Dynamics, Institute of Atmospheric Physics, Chinese Academy of Sciences, Beijing 100029, China

List of supporting figures

Supporting Figure S1

Supporting Figure S2

Supporting Figure S3

Supporting Figure S4

Supporting Figure S5

Supporting Figure S6

Supporting Figure S7

Supporting Figure S8

Supporting Figure S9

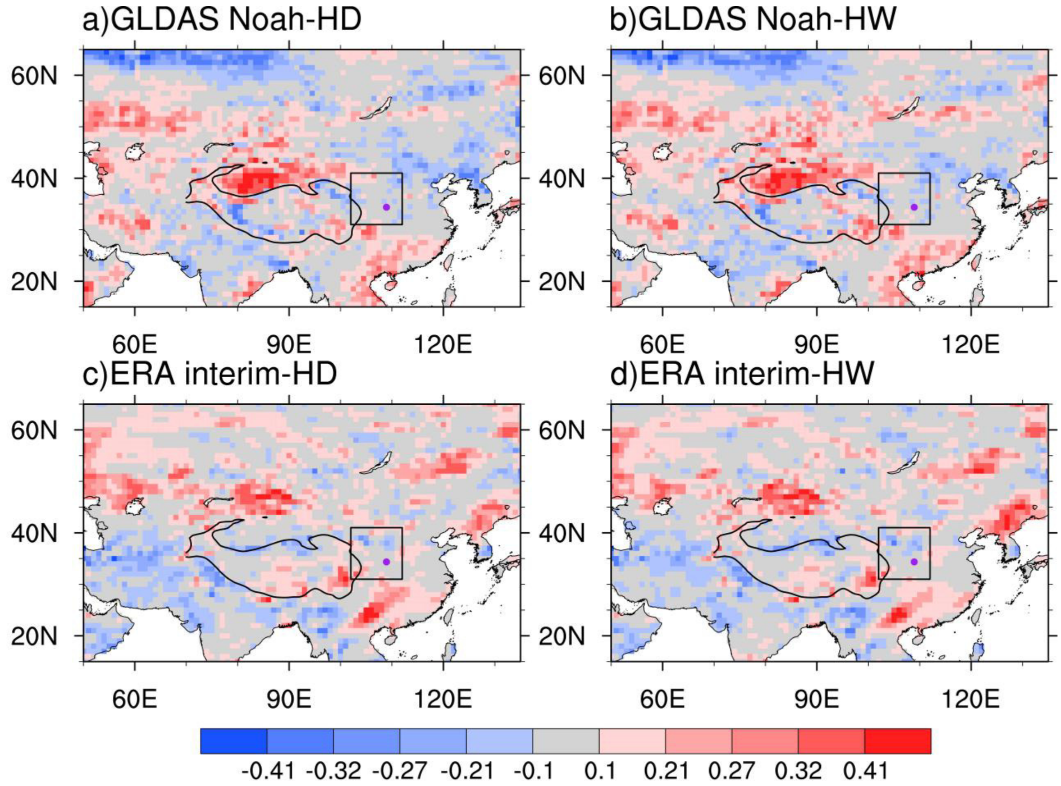

Figure S1. Correlation pattern of summer hot days (left panel) and heatwaves (right panel) averaged over the SRASR (source region of ancient Silk Road) enclosed by the black box with preceding spring soil moisture from (**a, b**) GLDAS-Noah and (**c, d**) ERA-interim for the period of 1979-2016. All data are detrended before correlation coefficients are calculated. The black solid line denotes topographic contour line of 3000 m. Correlations of ±0.27, ±0.32 and ±0.41 indicate the 90%, 95% and 99% significance levels. This map was generated with NCAR Command Language (NCL) version 6.3.0 (Boulder, Colorado: UCAR/NCAR/CISL/TDD. http://dx.doi.org/10.5065/D6WD3XH5).

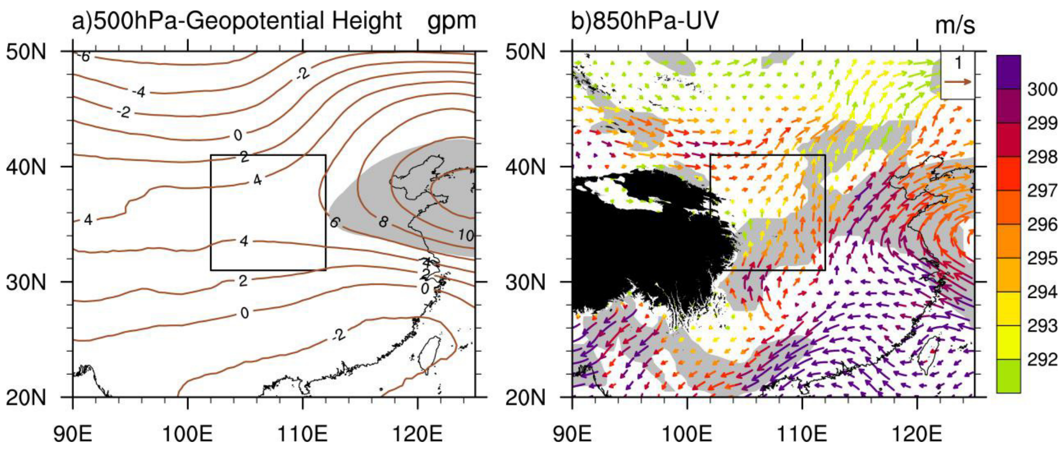

Figure S2. Composite differences between the ten warmest and ten coolest years of inter-annual component of spring soil temperature averaged over Central Asia in: (**a**) summer 500-hPa geopotential height, (**b**) summer 850-hPa horizontal wind vector. The colors of wind vector in (**b**) indicate 1979-2016 summer mean surface air temperature. All data are detrended. The shaded gray areas represent that the composite values are significant at the 90% confidence level. The black box denotes the SRASR (source region of ancient Silk Road). The black areas in (**b**) indicate that the elevations exceed 3000 m. This map was generated with NCAR Command Language (NCL) version 6.3.0 (Boulder, Colorado: UCAR/NCAR/CISL/TDD. http://dx.doi.org/10.5065/D6WD3XH5).

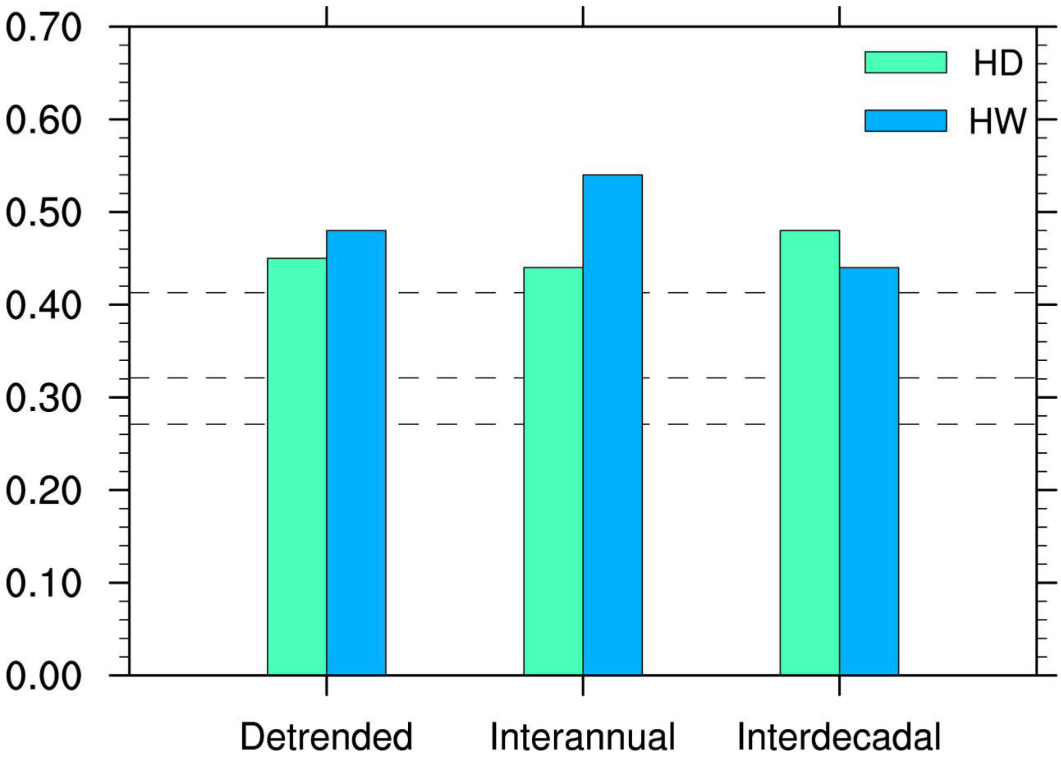

Figure S3. Correlation coefficients of spring sea surface temperature (SST) averaged over Northwest Atlantic (the key region of SST) with summer hot days (HD) and heatwaves (HW) averaged over the SRASR (source region of ancient Silk Road) for the period of 1979-2016: (left column) detrended time series; (middle column) inter-annual components of detrended time series; (right column) inter-decadal components of detrended time series. Correlations of ±0.27, ±0.32 and ±0.41 indicate the 90%, 95% and 99% significance levels (the dash lines).

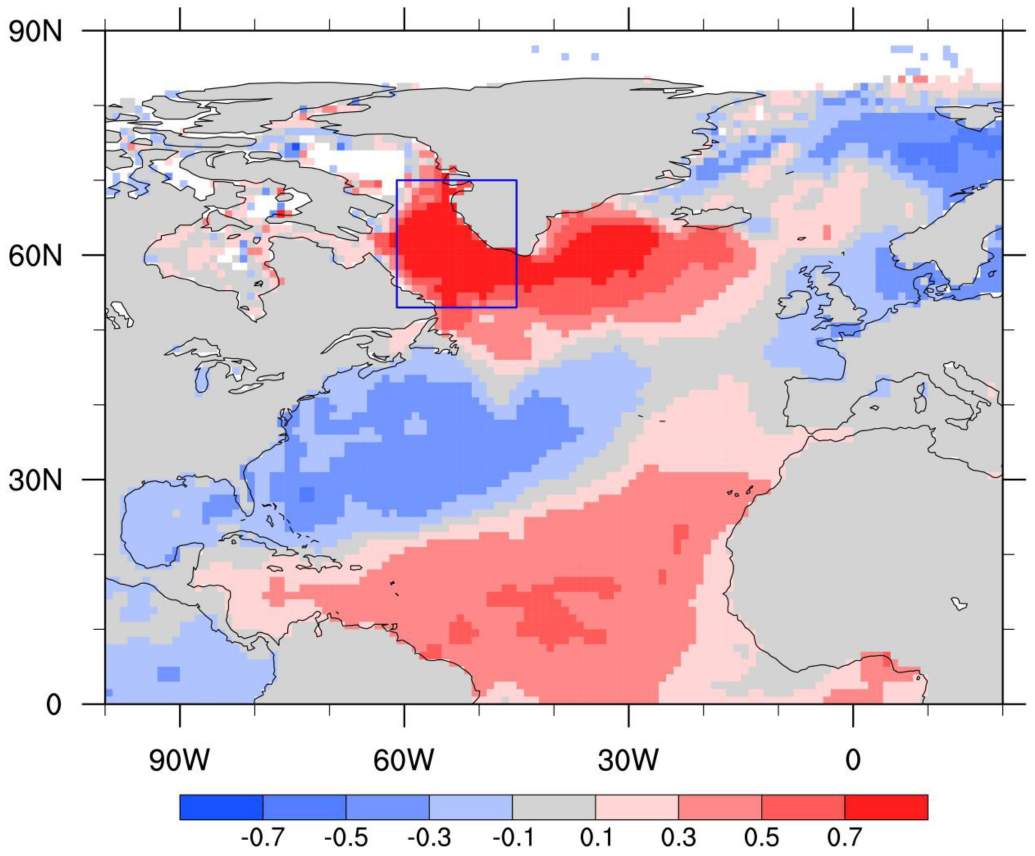

Figure S4. Correlation pattern of spring sea surface temperature (SST) averaged over the Northwest Atlantic (key region of SST) enclosed by the blue box with spring sea surface temperature (SST) for the period of 1979-2016. All data are detrended before the correlation coefficients are calculated. This map was generated with NCAR Command Language (NCL) version 6.3.0 (Boulder, Colorado: UCAR/NCAR/CISL/TDD. http://dx.doi.org/10.5065/D6WD3XH5).

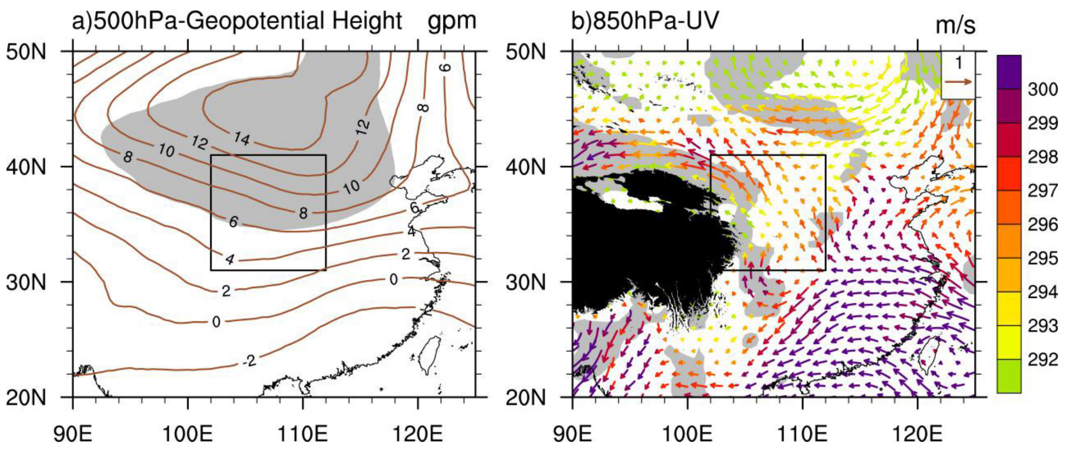

Figure S5. Composite differences between the ten warmest and ten coolest years of inter-annual component of spring sea surface temperature (SST) averaged over Northwest Atlantic in: (**a**) summer 500-hPa geopotential height, (**b**) summer 850-hPa horizontal wind vector. The colors of wind vector in (**b**) indicate 1979-2016 summer mean surface air temperature. All data are detrended. The shaded gray areas represent that the composite values are significant at the 90% confidence level. The black box denotes the SRASR (source region of ancient Silk Road). The black areas in (**b**) indicate that the elevations exceed 3000 m. This map was generated with NCAR Command Language (NCL) version 6.3.0 (Boulder, Colorado: UCAR/NCAR/CISL/TDD. http://dx.doi.org/10.5065/D6WD3XH5).

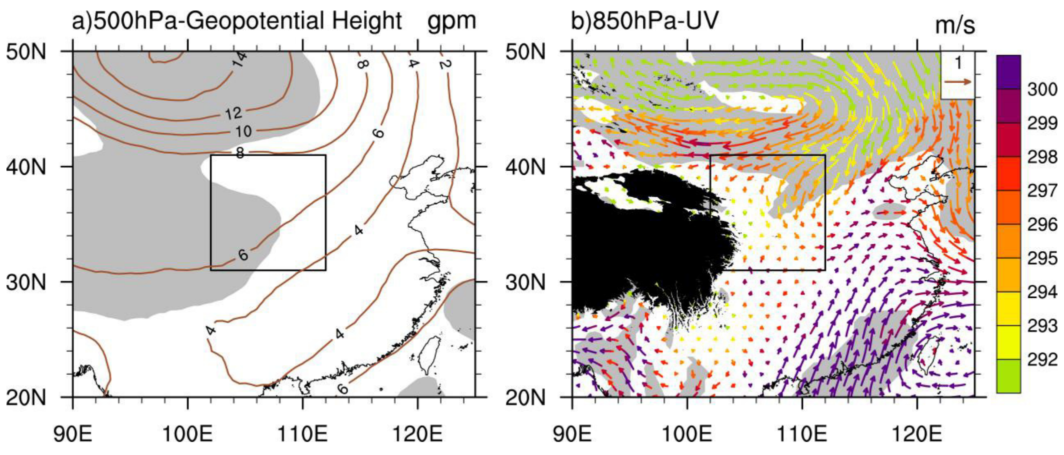

Figure S6. Composite differences between the ten highest and ten lowest years of inter-decadal component of the spring EP-NP pattern in: (**a**) summer 500-hPa geopotential height, (**b**) summer 850-hPa horizontal wind vector. The colors of wind vector in (**b**) indicate 1979-2016 summer mean surface air temperature. All data are detrended. The shaded gray areas represent that the composite values are significant at the 90% confidence level. The black box denotes the SRASR (source region of ancient Silk Road). The black areas in (**b**) indicate that the elevations exceed 3000 m. This map was generated with NCAR Command Language (NCL) version 6.3.0 (Boulder, Colorado: UCAR/NCAR/CISL/TDD. http://dx.doi.org/10.5065/D6WD3XH5).

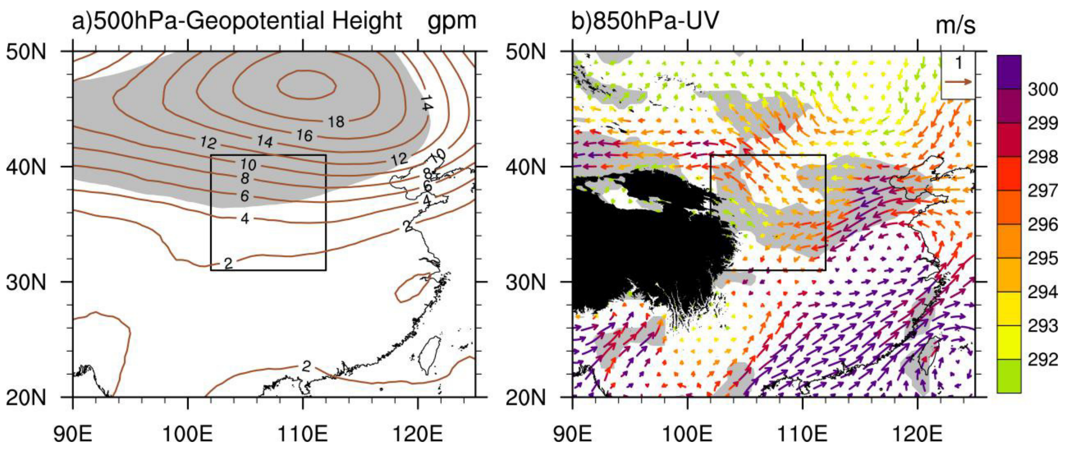

Figure S7. Composite differences between the ten highest and ten lowest years of inter-decadal component of the spring AMO in: (**a**) summer 500-hPa geopotential height, (**b**) summer 850-hPa horizontal wind vector. The colors of wind vector in (**b**) indicate 1979-2016 summer mean surface air temperature. All data are detrended. The shaded gray areas represent that the composite values are significant at the 90% confidence level. The black box denotes the SRASR (source region of ancient Silk Road). The black areas in (**b**) indicate that the elevations exceed 3000m. This map was generated with NCAR Command Language (NCL) version 6.3.0 (Boulder, Colorado: UCAR/NCAR/CISL/TDD. http://dx.doi.org/10.5065/D6WD3XH5).

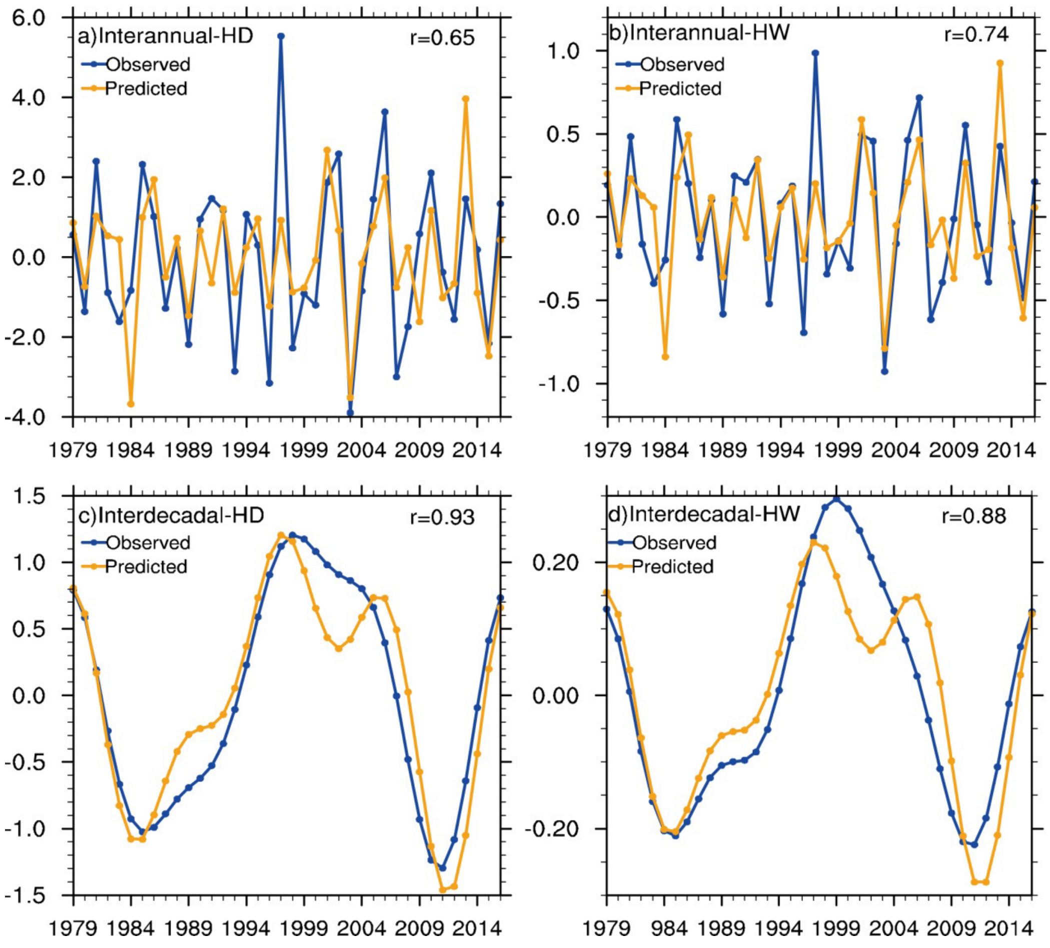

Figure S8. Time series of observed and predicted inter-annual (upper panel) and inter-decadal (lower panel) components of detrended summer hot temperature extremes averaged over the SRASR (source region of ancient Silk Road) for the period of 1979-2016: (**a, c**) hot days, (**b, d**) heatwaves. The correlation coefficients between the observations and predictions are also shown.

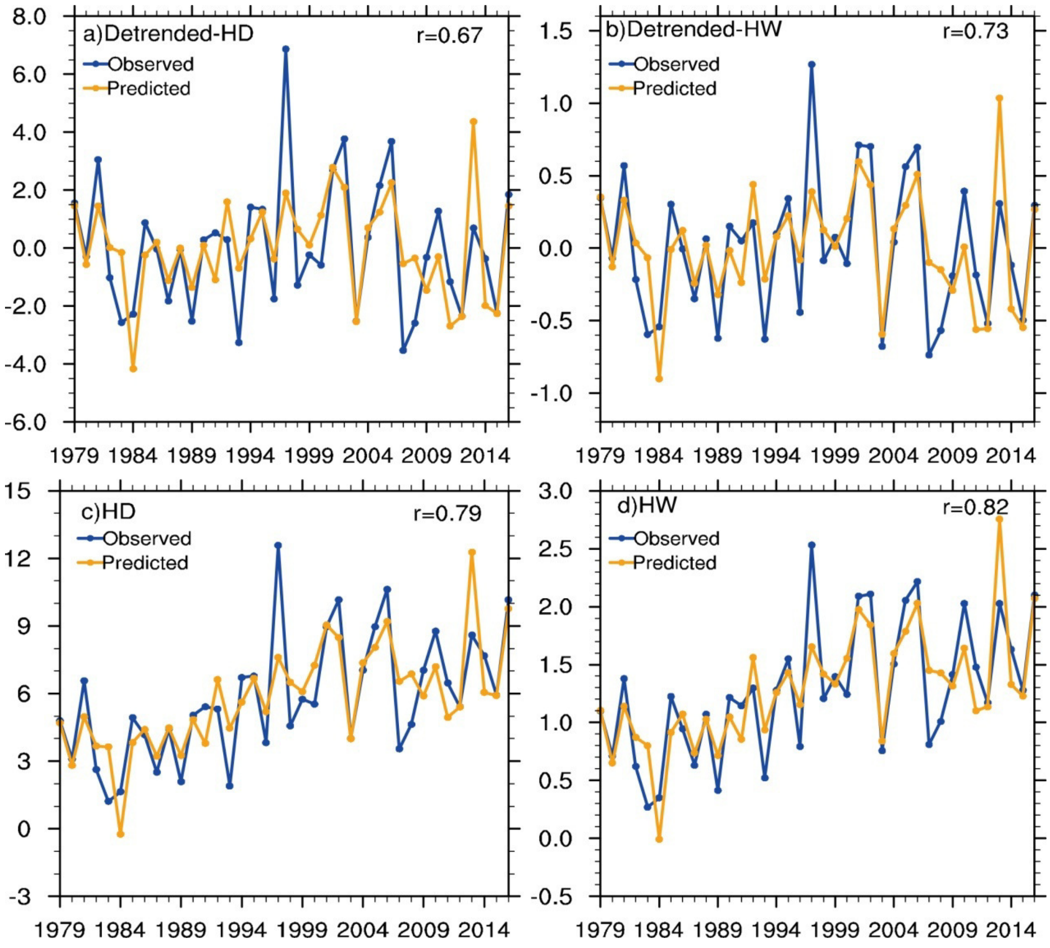

Figure S9. Time series of observed and predicted summer hot temperature extremes averaged over the SRASR (source region of ancient Silk Road) without (upper panel) and with (lower panel) linear trends included for the period of 1979-2016: (**a, c**) hot days, (**b, d**) heatwaves. The predictions are made by using March-April averaged predictors. The correlation coefficients between the observations and predictions are also shown.
